# (*E*)-1-(2,4,6-Trimeth­oxy­phen­yl)pent-1-en-3-one

**DOI:** 10.1107/S1600536810034641

**Published:** 2010-09-11

**Authors:** Alain Collas, Frank Blockhuys

**Affiliations:** a Department of Chemistry, University of Antwerp, Universiteitsplein 1, B-2610 Wilrijk, Belgium

## Abstract

The title compound, C_14_H_18_O_4_, was obtained unintentionally as the major product of an attempted synthesis of (*E*,*E*)-2,5-bis­[2-(2,4,6-trimeth­oxy­phen­yl)ethen­yl]pyrazine. The crystal packing features layers based on two weak C—H⋯O hydrogen bonds involving the O atom of the carbonyl group and two O_meth­oxy_⋯C_meth­oxy_ inter­actions [3.109 (2) Å]. The sheets are inter­connected *via* meth­oxy–meth­oxy dimers and C—H⋯π inter­actions.

## Related literature

For related compounds containing the Ph—CH=CH—CO— fragment, see: Zhang *et al.* (2008 ▶); Degen & Bolte (1999 ▶); Zonouzi *et al.* (2009 ▶); Wang *et al.* (2005 ▶). For π-bridged donor–acceptor–donor systems as candidates for organic light-emitting diodes and their non-linear optical properties, see Liu *et al.* (2001 ▶); Grimsdale *et al.* (1997 ▶); Chemla (1987 ▶). For a description of the Cambridge Structural Database, see: Allen (2002 ▶).
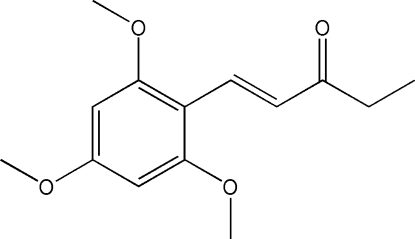


## Experimental

### 

#### Crystal data

C_14_H_18_O_4_
*M* *_r_* = 250.28
Triclinic, 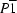

*a* = 6.8626 (8) Å
*b* = 8.297 (1) Å
*c* = 12.068 (2) Å
α = 71.96 (1)°
β = 84.28 (1)°
γ = 84.90 (1)°
*V* = 648.88 (15) Å^3^
*Z* = 2
Mo *K*α radiation
μ = 0.09 mm^−1^
*T* = 293 K
0.3 × 0.24 × 0.18 mm

#### Data collection

Enraf–Nonius CAD-4 diffractometer
4732 measured reflections
2366 independent reflections
1589 reflections with > 2/s(*I*)
*R*_int_ = 0.020
3 standard reflections every 60 min  intensity decay: none

#### Refinement

*R*[*F* ^2^ > 2σ(*F* ^2^)] = 0.037
*wR*(*F* ^2^) = 0.106
*S* = 1.03
2366 reflections
166 parameters
H-atom parameters constrained
Δρ_max_ = 0.14 e Å^−3^
Δρ_min_ = −0.17 e Å^−3^

### 

Data collection: *CAD-4 EXPRESS* (Enraf–Nonius, 1994 ▶); cell refinement: *CAD-4 EXPRESS*; data reduction: *XCAD4* (Harms & Wocadlo, 1996 ▶); program(s) used to solve structure: *SHELXS97* (Sheldrick, 2008 ▶); program(s) used to refine structure: *SHELXL97* (Sheldrick, 2008 ▶); molecular graphics: *ORTEP-3 for Windows* (Farrugia, 1997 ▶); software used to prepare material for publication: *WinGX* (Farrugia, 1999 ▶), *Mercury* (Macrae *et al.*, 2008 ▶) and *PLATON* (Spek, 2009 ▶).

## Supplementary Material

Crystal structure: contains datablocks I, global. DOI: 10.1107/S1600536810034641/zl2296sup1.cif

Structure factors: contains datablocks I. DOI: 10.1107/S1600536810034641/zl2296Isup2.hkl

Additional supplementary materials:  crystallographic information; 3D view; checkCIF report

## Figures and Tables

**Table 1:** Hydrogen-bond geometry (Å, °) *Cg* is the centroid of the C1–C6 ring.

*D*—H⋯*A*	*D*—H	H⋯*A*	*D*⋯*A*	*D*—H⋯*A*
C5—H5⋯O4^i^	0.93	2.59	3.517 (2)	177
C31—H31*C*⋯O4^i^	0.96	2.70	3.286 (2)	120
C21—H21*C*⋯O2^ii^	0.96	2.76	3.419 (2)	127
C11—H11*A*⋯O4^iii^	0.96	2.75	3.557 (2)	142
C12—H12*C*⋯O4^iv^	0.96	2.76	3.706 (2)	167
C10—H10*B*⋯*Cg*^v^	0.97	2.77	3.59	142

Symmetry codes: (i) 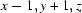; (ii) 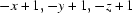; (iii) 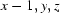; (iv) 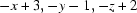; (v) 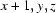.

**Fig. 1.**
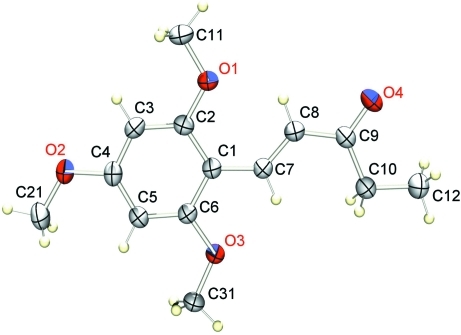
: Molecular structure of the title compound showing the numbering scheme. Displacement ellipsoids are drawn at the 50% probability level; hydrogen atoms are represented by spheres with an arbitrary radius.

**Fig. 2.**
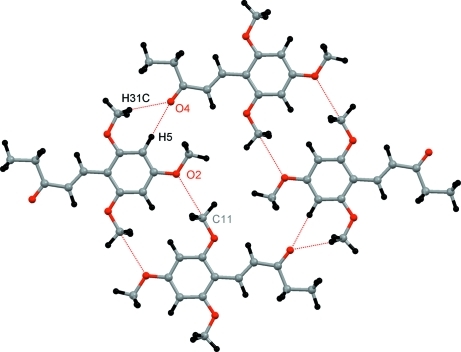
: Sheets formed by weak hydrogen bonds and Omethoxy···Cmethoxy interactions.

**Fig. 3.**
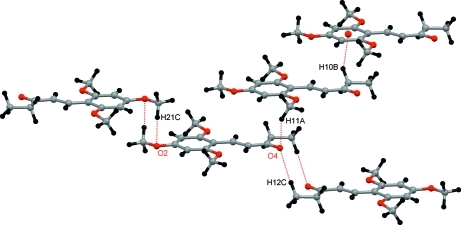
: Interactions responsible for the stabilization of the crystal packing in the direction perpendicular to the the generated sheets.

## supplementary crystallographic information

### Comment

π-Bridged donor-acceptor-donor (A—D—A) systems are promising candidates for electronic applications such as organic light-emitting diodes (Liu *et al.*, 2001; Grimsdale *et al.*, 1997), as they are expected to have electronic properties similar to those of conventional OPV-type systems but with a red-shifted emission spectrum. Moreover, due to their high degree of conjugation, these A—D—A oligomers are also excellent candidates for organic non-linear optic (NLO) media with a high second-order hyperpolarizability, γ (Chemla, 1987). In an attempt to synthesize the A—D—A system *E*,*E*-2,5-bis[2-(2,4,6-trimethoxyphenyl)ethenyl]pyrazine from dimethylpyrazine and the relevant benzaldehyde under standard condensation conditions, (*E*)-1-(2,4,6-trimethoxyphenyl)pent-1-en-3-one (Fig. 1) was obtained as the major product. In this compound the C=C bond is not disordered, in contrast to what is the case in the 3-methoxy-4-acetoxyphenyl derivative YODGOO (Zhang *et al.*, 2008) and the molecule adopts the *anti* conformation indicating that there are no energetically beneficial intermolecular contacts favouring the *syn* conformation as in the unsubstituted DIBNEH (Degen & Bolte, 1999). The title compound displays two weak intramolecular hydrogen bonds involving the methoxy groups in the *ortho* positions of the phenyl ring, one in a five- and one in a six-membered ring configuration. In contrast, in the 2-hydroxy-5-bromophenyl derivative NORGOR (Zonouzi *et al.*, 2009) a less stable six-membered ring configuration is observed due to the competing strong intermolecular O—H···O hydrogen bond with an adjacent molecule. In the 2-hydroxyphenyl derivative FONKEZ (Wang *et al.*, 2005), the more favourable five-membered ring configuration is also seen. The packing of the title compound is determined in first instance by contacts between the methoxy groups. Two molecules (symmetry-related *via* an inversion centre) are connected into a dimer involving O2 and C11 of the methoxy groups in the 2- and 4-positions [C11···O2^i^, 3.109 (2) Å, 178.33 (12)°, symm. code i = 1 - *x*, -*y*, 1 - *z*] (Fig. 2); note that the C···O—C angle is almost linear. Sheets are then generated through two weak hydrogen bonds involving H5 (Table 1, entry 1) and H31C (entry 2) contacting the oxygen atom of the carbonyl group (O4). These sheets are then interconnected by four additional weak hydrogen bonds (Fig. 3): H21C and O2 are involved in a second dimer formation (entry 3), H11A and H12C contact the oxygen atom of the carbonyl (O4, entries 4 and 5) and H10B of the methylene group next to the carbonyl group generates a CH···π interaction with a nearby phenyl ring (entry 6).

### Experimental

A solution of sodium (1.0 g, 0.04 mol) in ethanol (50 ml) was added dropwise to a solution of 2,4,6-trimethoxybenzaldehyde (5.6 g, 0.04 mol) and 2,5-dimethylpyrazine (2.2 g, 0.02 mol) in ethanol (150 ml) at room temperature and the reaction mixture was heated under reflux for 4 h. The resulting fluorescent yellow solution was poured into 500 ml of ice water and the precipitate was filtered off and isomerized to the all-E form in *p*-xylene with a catalytic amount of iodine. Crystals suitable for X-ray diffraction were grown by slow evaporation of a THF solution. The yield was 2.3 g (46%). M.p. (uncorrected) 401 K. ^1^H NMR (CDCl_3_, 400 MHz, TMS): δ 7.63 (td, 8 and 0.4 Hz, H5), 8.18 (ddd, 8, 2 and 1 Hz, H6), 8.27 (ddd, 8, 2 and 1 Hz, H4), 8.62 (td, 2 and 0.4 Hz, H2), H4 and H6 appear to be magnetically equivalent. ^13^C NMR (CDCl_3_, 100 MHz, TMS): δ 121.59 (C2), 124.54 (C5), 130.24 (C6), 132.32 (C4), 142.46 (C1), 148.53 (C3).

### Refinement

Hydrogen atoms were placed in calculated positions and refined as riding with C—H distances of 0.93 Å.

### Crystal data

**Table:**

C_14_H_18_O_4_	*Z* = 2
*M**_r_* = 250.28	*F*(000) = 268
Triclinic, *P*1	*D*_x_ = 1.281 Mg m^−^^3^
Hall symbol: -P 1	Melting point: 401 K
*a* = 6.8626 (8) Å	Mo *K*α radiation, λ = 0.71073 Å
*b* = 8.297 (1) Å	Cell parameters from 25 reflections
*c* = 12.068 (2) Å	θ = 5.8–10.7°
α = 71.96 (1)°	µ = 0.09 mm^−^^1^
β = 84.28 (1)°	*T* = 293 K
γ = 84.90 (1)°	Prism, yellow
*V* = 648.88 (15) Å^3^	0.3 × 0.24 × 0.18 mm

### Data collection

**Table:**

Enraf–Nonius CAD-4 diffractometer	*R*_int_ = 0.020
Radiation source: fine-focus sealed tube	θ_max_ = 25.3°, θ_min_ = 1.8°
graphite	*h* = −8→8
non–profiled ω/2θ scans	*k* = −9→9
4732 measured reflections	*l* = −14→14
2366 independent reflections	3 standard reflections every 60 min
1589 reflections with > 2/s(*I*)	intensity decay: none

### Refinement

**Table:**

Refinement on *F*^2^	Primary atom site location: structure-invariant direct methods
Least-squares matrix: full	Secondary atom site location: difference Fourier map
*R*[*F*^2^ > 2σ(*F*^2^)] = 0.037	Hydrogen site location: inferred from neighbouring sites
*wR*(*F*^2^) = 0.106	H-atom parameters constrained
*S* = 1.02	*w* = 1/[σ^2^(*F*_o_^2^) + (0.0495*P*)^2^ + 0.1212*P*] where *P* = (*F*_o_^2^ + 2*F*_c_^2^)/3
2366 reflections	(Δ/σ)_max_ < 0.001
166 parameters	Δρ_max_ = 0.14 e Å^−^^3^
0 restraints	Δρ_min_ = −0.17 e Å^−^^3^

### Special details

**Table:**

Geometry. All e.s.d.'s (except the e.s.d. in the dihedral angle between two l.s. planes) are estimated using the full covariance matrix. The cell e.s.d.'s are taken into account individually in the estimation of e.s.d.'s in distances, angles and torsion angles; correlations between e.s.d.'s in cell parameters are only used when they are defined by crystal symmetry. An approximate (isotropic) treatment of cell e.s.d.'s is used for estimating e.s.d.'s involving l.s. planes.
Refinement. Refinement of *F*^2^ against ALL reflections. The weighted *R*-factor *wR* and goodness of fit *S* are based on *F*^2^, conventional *R*-factors *R* are based on *F*, with *F* set to zero for negative *F*^2^. The threshold expression of *F*^2^ > σ(*F*^2^) is used only for calculating *R*-factors(gt) *etc*. and is not relevant to the choice of reflections for refinement. *R*-factors based on *F*^2^ are statistically about twice as large as those based on *F*, and *R*- factors based on ALL data will be even larger.

### Fractional atomic coordinates and isotropic or equivalent isotropic displacement parameters (Å^2^)

**Table:**

	*x*	*y*	*z*	*U*_iso_*/*U*_eq_	
C12	1.6553 (3)	−0.2420 (2)	0.97422 (19)	0.0572 (5)	
H12A	1.6978	−0.1867	1.0259	0.086*	
H12B	1.7585	−0.2453	0.9149	0.086*	
H12C	1.6235	−0.3558	1.0179	0.086*	
O2	0.41445 (17)	0.31488 (16)	0.60482 (12)	0.0544 (4)	
O1	0.90913 (18)	−0.12493 (15)	0.63577 (11)	0.0498 (3)	
O3	0.95452 (17)	0.26141 (15)	0.84353 (11)	0.0508 (4)	
C2	0.8301 (2)	0.0153 (2)	0.66303 (15)	0.0389 (4)	
C1	0.9334 (2)	0.0684 (2)	0.73980 (14)	0.0373 (4)	
O4	1.4852 (2)	−0.34752 (17)	0.81476 (13)	0.0675 (4)	
C3	0.6593 (2)	0.1019 (2)	0.61907 (15)	0.0434 (4)	
H3	0.5942	0.0657	0.5679	0.052*	
C7	1.1125 (2)	−0.0161 (2)	0.79054 (14)	0.0383 (4)	
H7	1.1607	0.0323	0.8414	0.046*	
C6	0.8527 (2)	0.2139 (2)	0.76858 (14)	0.0375 (4)	
C8	1.2193 (2)	−0.1515 (2)	0.77646 (16)	0.0440 (4)	
H8	1.1771	−0.2042	0.7262	0.053*	
C5	0.6805 (2)	0.3019 (2)	0.72569 (15)	0.0405 (4)	
H5	0.6307	0.3975	0.7462	0.049*	
C9	1.3985 (2)	−0.2232 (2)	0.83457 (15)	0.0414 (4)	
C21	0.3216 (3)	0.4540 (3)	0.63919 (19)	0.0590 (5)	
H21A	0.2956	0.4200	0.7226	0.088*	
H21B	0.2003	0.4891	0.6027	0.088*	
H21C	0.4061	0.5469	0.6155	0.088*	
C10	1.4756 (3)	−0.1447 (2)	0.91735 (15)	0.0427 (4)	
H10A	1.3731	−0.1395	0.9778	0.051*	
H10B	1.5081	−0.0292	0.8748	0.051*	
C4	0.5856 (2)	0.2428 (2)	0.65167 (15)	0.0408 (4)	
C31	0.8968 (3)	0.4174 (2)	0.86731 (18)	0.0548 (5)	
H31A	0.8984	0.5088	0.7953	0.082*	
H31B	0.9863	0.4371	0.9174	0.082*	
H31C	0.7667	0.4115	0.9053	0.082*	
C11	0.8077 (3)	−0.1881 (2)	0.56160 (17)	0.0535 (5)	
H11A	0.6802	−0.2198	0.5982	0.080*	
H11B	0.8809	−0.2856	0.5486	0.080*	
H11C	0.7938	−0.1015	0.4882	0.080*	

### Atomic displacement parameters (Å^2^)

**Table:**

	*U*^11^	*U*^22^	*U*^33^	*U*^12^	*U*^13^	*U*^23^
C12	0.0540 (12)	0.0567 (12)	0.0682 (13)	0.0125 (9)	−0.0247 (10)	−0.0277 (10)
O2	0.0432 (7)	0.0571 (8)	0.0729 (9)	0.0161 (6)	−0.0281 (6)	−0.0321 (7)
O1	0.0480 (7)	0.0507 (7)	0.0651 (8)	0.0121 (6)	−0.0228 (6)	−0.0368 (7)
O3	0.0475 (7)	0.0496 (7)	0.0707 (9)	0.0177 (6)	−0.0281 (6)	−0.0391 (7)
C2	0.0383 (9)	0.0378 (9)	0.0456 (10)	0.0026 (7)	−0.0072 (7)	−0.0201 (8)
C1	0.0339 (9)	0.0384 (9)	0.0434 (10)	0.0034 (7)	−0.0085 (7)	−0.0177 (8)
O4	0.0663 (9)	0.0600 (9)	0.0944 (11)	0.0310 (7)	−0.0347 (8)	−0.0504 (8)
C3	0.0412 (10)	0.0478 (10)	0.0495 (11)	0.0017 (8)	−0.0167 (8)	−0.0241 (9)
C7	0.0358 (9)	0.0394 (9)	0.0452 (10)	0.0036 (7)	−0.0106 (7)	−0.0199 (8)
C6	0.0355 (9)	0.0389 (9)	0.0431 (9)	0.0025 (7)	−0.0095 (7)	−0.0190 (8)
C8	0.0435 (10)	0.0436 (10)	0.0538 (11)	0.0070 (8)	−0.0168 (8)	−0.0261 (9)
C5	0.0385 (9)	0.0364 (9)	0.0498 (10)	0.0063 (7)	−0.0096 (8)	−0.0181 (8)
C9	0.0405 (9)	0.0371 (9)	0.0497 (11)	0.0073 (8)	−0.0087 (8)	−0.0190 (8)
C21	0.0445 (11)	0.0633 (13)	0.0740 (14)	0.0208 (9)	−0.0200 (10)	−0.0298 (11)
C10	0.0436 (10)	0.0390 (9)	0.0485 (10)	0.0055 (8)	−0.0107 (8)	−0.0175 (8)
C4	0.0330 (9)	0.0431 (10)	0.0463 (10)	0.0042 (7)	−0.0098 (8)	−0.0132 (8)
C31	0.0526 (11)	0.0507 (11)	0.0773 (14)	0.0156 (9)	−0.0255 (10)	−0.0415 (11)
C11	0.0562 (11)	0.0568 (12)	0.0629 (12)	0.0039 (9)	−0.0177 (9)	−0.0385 (10)

### Geometric parameters (Å, °)

**Table:**

C12—C10	1.514 (2)	C7—H7	0.9300
C12—H12A	0.9600	C6—C5	1.390 (2)
C12—H12B	0.9600	C8—C9	1.463 (2)
C12—H12C	0.9600	C8—H8	0.9300
O2—C4	1.3620 (19)	C5—C4	1.381 (2)
O2—C21	1.423 (2)	C5—H5	0.9300
O1—C2	1.3584 (19)	C9—C10	1.508 (2)
O1—C11	1.4287 (19)	C21—H21A	0.9600
O3—C6	1.3629 (19)	C21—H21B	0.9600
O3—C31	1.4253 (19)	C21—H21C	0.9600
C2—C3	1.382 (2)	C10—H10A	0.9700
C2—C1	1.412 (2)	C10—H10B	0.9700
C1—C6	1.409 (2)	C31—H31A	0.9600
C1—C7	1.452 (2)	C31—H31B	0.9600
O4—C9	1.2206 (19)	C31—H31C	0.9600
C3—C4	1.385 (2)	C11—H11A	0.9600
C3—H3	0.9300	C11—H11B	0.9600
C7—C8	1.332 (2)	C11—H11C	0.9600
			
C10—C12—H12A	109.5	O4—C9—C8	118.99 (15)
C10—C12—H12B	109.5	O4—C9—C10	120.39 (15)
H12A—C12—H12B	109.5	C8—C9—C10	120.62 (14)
C10—C12—H12C	109.5	O2—C21—H21A	109.5
H12A—C12—H12C	109.5	O2—C21—H21B	109.5
H12B—C12—H12C	109.5	H21A—C21—H21B	109.5
C4—O2—C21	118.09 (13)	O2—C21—H21C	109.5
C2—O1—C11	118.44 (13)	H21A—C21—H21C	109.5
C6—O3—C31	119.00 (12)	H21B—C21—H21C	109.5
O1—C2—C3	122.66 (14)	C9—C10—C12	113.02 (14)
O1—C2—C1	115.88 (14)	C9—C10—H10A	109.0
C3—C2—C1	121.46 (14)	C12—C10—H10A	109.0
C6—C1—C2	116.34 (14)	C9—C10—H10B	109.0
C6—C1—C7	118.79 (15)	C12—C10—H10B	109.0
C2—C1—C7	124.87 (14)	H10A—C10—H10B	107.8
C2—C3—C4	119.62 (15)	O2—C4—C5	124.12 (15)
C2—C3—H3	120.2	O2—C4—C3	114.18 (14)
C4—C3—H3	120.2	C5—C4—C3	121.69 (15)
C8—C7—C1	130.83 (16)	O3—C31—H31A	109.5
C8—C7—H7	114.6	O3—C31—H31B	109.5
C1—C7—H7	114.6	H31A—C31—H31B	109.5
O3—C6—C5	121.97 (14)	O3—C31—H31C	109.5
O3—C6—C1	115.08 (13)	H31A—C31—H31C	109.5
C5—C6—C1	122.94 (14)	H31B—C31—H31C	109.5
C7—C8—C9	124.60 (15)	O1—C11—H11A	109.5
C7—C8—H8	117.7	O1—C11—H11B	109.5
C9—C8—H8	117.7	H11A—C11—H11B	109.5
C4—C5—C6	117.93 (15)	O1—C11—H11C	109.5
C4—C5—H5	121.0	H11A—C11—H11C	109.5
C6—C5—H5	121.0	H11B—C11—H11C	109.5
			
C11—O1—C2—C3	−2.0 (2)	C7—C1—C6—C5	−179.07 (16)
C11—O1—C2—C1	177.92 (16)	C1—C7—C8—C9	−179.80 (17)
O1—C2—C1—C6	179.96 (15)	O3—C6—C5—C4	−178.96 (16)
C3—C2—C1—C6	−0.1 (2)	C1—C6—C5—C4	0.0 (3)
O1—C2—C1—C7	−0.5 (3)	C7—C8—C9—O4	−179.72 (18)
C3—C2—C1—C7	179.42 (17)	C7—C8—C9—C10	0.1 (3)
O1—C2—C3—C4	179.20 (16)	O4—C9—C10—C12	−3.5 (3)
C1—C2—C3—C4	−0.8 (3)	C8—C9—C10—C12	176.69 (17)
C6—C1—C7—C8	−178.60 (19)	C21—O2—C4—C5	−3.7 (3)
C2—C1—C7—C8	1.9 (3)	C21—O2—C4—C3	176.19 (16)
C31—O3—C6—C5	−7.9 (3)	C6—C5—C4—O2	178.94 (16)
C31—O3—C6—C1	173.02 (16)	C6—C5—C4—C3	−0.9 (3)
C2—C1—C6—O3	179.50 (15)	C2—C3—C4—O2	−178.58 (16)
C7—C1—C6—O3	0.0 (2)	C2—C3—C4—C5	1.3 (3)
C2—C1—C6—C5	0.5 (3)		

### Hydrogen-bond geometry (Å, °)

**Table:**

Cg is the centroid of the C1–C6 ring.

**Table:**

*D*—H···*A*	*D*—H	H···*A*	*D*···*A*	*D*—H···*A*
C5—H5···O4^i^	0.93	2.59	3.517 (2)	177
C31—H31C···O4^i^	0.96	2.70	3.286 (2)	120
C21—H21C···O2^ii^	0.96	2.76	3.419 (2)	127
C11—H11A···O4^iii^	0.96	2.75	3.557 (2)	142
C12—H12C···O4^iv^	0.96	2.76	3.706 (2)	167
C10—H10B···Cg^v^	0.97	2.77	3.59	142

Symmetry codes: (i) *x*−1, *y*+1, *z*; (ii) −*x*+1, −*y*+1, −*z*+1; (iii) *x*−1, *y*, *z*; (iv) −*x*+3, −*y*−1, −*z*+2; (v) *x*+1, *y*, *z*.
